# Editorial: Real-world implementation of the biopsychosocial approach to healthcare: Pragmatic approaches, success stories and lessons learned

**DOI:** 10.3389/fpsyt.2022.1026415

**Published:** 2022-09-13

**Authors:** Marsha Nicole Wittink, Tziporah Rosenberg, Christiane Waller, Peiyuan Qiu, Susan McDaniel

**Affiliations:** ^1^Department of Psychiatry, University of Rochester School of Medicine and Dentistry, Rochester, NY, United States; ^2^Department of Family Medicine University of Rochester School of Medicine and Dentistry, Rochester, NY, United States; ^3^Universitätsklinik für Psychiatrie und Psychotherapie, Paracelsus Medizinischen Privatuniversität Nuremberg, Nuremberg, Germany; ^4^West China School of Public Health, Sichuan University, Chengdu, China

**Keywords:** biopsychosocial, COVID, globalization, medical mistrust, social determinants of health, context, team based, healthcare [D000067575]

In 1977, George Engel's landmark paper (1) challenged the medical community to re-think how healthcare could be conceptualized, taught and practiced. Engel–an internist and psychoanalyst who spent much of his career in the Department of Psychiatry at the University of Rochester–pointed to the importance of widening our view from the still-important biomedical aspects of disease and illness to also examine psychological and social factors of influence. He was particularly concerned with the idea of reductionism (that illness can be attributed to one singular cause), and mind-body dualism (the notion that the mind and the body operate completely independently of one another). As a psychoanalyst, Engel was trained to focus on the role patients' beliefs, behaviors and relationships played in their experiences of health and disease. Perhaps this also allowed him to become more acutely aware of how clinicians' belief systems could limit conceptualization of disease. He wrote, “The historical fact we have to face is that in modern Western society, biomedicine not only has provided a basis for the scientific study of disease, it has also become our own culturally specific perspective about disease, that is, our folk model.” He set out to encourage clinicians to embrace a more comprehensive understanding of the psychosocial aspects of health that can influence how health is experienced as well as the course of disease and the trajectory of recovery. The timing of Engel's paper was ideal; there was a growing movement of concern that medical care was becoming increasingly specialized and de-humanized (2). The biopsychosocial (BPS) approach provided a helpful framework for the time.

Today, many in healthcare are once again reconceptualizing the pitfalls of the still predominant biomedical approach to healthcare. The COVID pandemic has reinforced systemic health inequities (3) and public mistrust of mainstream healthcare is on the rise (4). At the same time, surmounting evidence over the last several decades shows that psychosocial stressors in early childhood have physiological and immune modulating effects on the body (5, 6) and that structural determinants reinforce health inequities and exacerbate such stressors (7). Hence, the value of the BPS approach remains even more relevant today, perhaps with greater attention to the role of the nuances of the social component of the BPS. The BPS framework is also relevant in a health system that increasingly recognizes the importance of health care teams to expand the scope of expertise of those in healthcare to the psychological and social realms, as well as to address the dramatically increasing burden and burnout of physicians and nurses (8).

With this Research Topic, we sought to survey how the BPS approach is being conceptualized, adapted and improved in the current climate of healthcare challenges internationally. We invited authors to tell us how they are using the BPS pragmatically in clinical and community settings, and we encouraged them to tell us about the ways in which they are adding to the framework and working across disciplines to address each component. We found that clinicians and researchers across the world are actively thinking about and implementing components of the BPS framework, adapting the approach to address the challenges they face caring for an ever increasingly complex set of concerns, and continuing to define the BPS framework to incorporate important changes in the way healthcare is being delivered and experienced today. We have categorized the manuscripts in this special edition to highlight four main cross-cutting themes: 1. Conceptualization and additions to the BPS approach; 2. The use of the BPS to approach to address health-related issues with etiologies and treatments that reach beyond the historically defined (though often arbitrary) biomedical boundaries of medical care; 3. Description of the opportunities and challenges of interprofessional teamwork to actualize components of the BPS framework; and 4. Reports of educational innovations related to the BPS approach. Below we describe each theme and discuss key insights from the papers in this Research Topic.

## Conceptualization and additions to BPS approach

The BPS approach is reflective of the general systems model that permeated physics, biology, and eventually the social sciences in the mid-20th century. While lauded for its “clinical merit,” the BPS approach has been criticized as “underdeveloped” as a scientific model due to its vagueness (9, 10). Nonetheless, the pragmatic tenets of attending to biological, psychological and social components of health are compelling, with many clinicians and researchers desiring to add specificity and theoretical rigor to how the BPS approach can be incorporated in practice (11), including the additions in this section. Several focus on the need to expand the BPS approach to address psychosocial components of health, such as the role of family and relational aspects of health. Wood et al. take up the challenge through their development of the Biobehavioral Family Model (BBFM), which identifies specific pathways by which family relationships impact disease activity through psychobiological mechanisms. Schwartz et al. review the literature and make a policy argument for systemic change in intensive care units to embrace “family centered care” so that “family members feel respected as valued members of the care team.” In their perspective paper Hiefner and Villareal similarly recommend adding a family-oriented, multidisciplinary approach to caring for patients and families after a miscarriage. Podgorski et al. take a slightly different tack, invoking the Socioecological Model with regard to dementia caregiving to consider the impact and context of illness within family relationships and social networks.

Each of the above papers add specificity to the psychosocial aspects of the BPS approach or address explicit frameworks for BPS implementation. In doing so, they invite us to question just how many aspects of a holistic model should be incorporated at any one time: should emotional and family factors be considered in addition to or separate from other interpersonal factors? Should cultural factors be included in addition to historical systems, and in which context? Ventres and Frankel address this line of questioning head-on, stating “the BPS model is not set in stone, but an inspiration for further integrating BPS concepts into practice.” Using the concepts of “add ons” and “add ins,” they suggest that clinicians can take personal ownership over the BPS approach, flexing and focusing as relevant.

## Using the BPS to develop new solutions to complex health challenges

The BPS approach may be particularly helpful in developing solutions to health-related challenges that have been limited by the arbitrary boundaries of healthcare settings where people often seek care. For example, Hou et al. use the BPS approach to consider the impact of migration and isolation on health needs of women affected by intimate partner violence in rural China. Guo et al. use the BPS approach to examine the relationship between adherence to traditional Chinese post-partum practices and post-partum depression and Duberstein et al. use the BPS approach to identify personal, psychological, social and family contexts that impact pre- and post-natal care utilization in the community. Van Orden et al. demonstrate ways in which the electronic health record can be leveraged to assess patient-reported outcomes (PROs) and thereby develop population-based strategies for addressing BPS needs in a health system. Chen et al. describe a layperson-delivered intervention using a BPS framework to address psychiatric support needs in low and middle-income countries suffering from lack of resources and specialized trained professionals. Schaefert et al. conducted a cross-country survey to assess the range of psychological and psychiatric consultation and liaison (CL) services that arose in the context of COVID. Using a BPS perspective, they consider their findings that CL services provide for healthcare workers and families in addition to patients and suggest ways of working with administration and across systems to bolster CL services. Köbler et al. use the BPS framework to discuss a new type of integrated medical- psychiatry unit targeted to patients with medical conditions (“somatically ill patients”) that otherwise limit their ability to receive focused treatment. Stoll et al. use the BPS framework in a study of psychiatrists' attitudes toward Palliative Psychiatry- a new approach to care for patients with severe mental illness which includes assessment of existential factors of care. By comparing attitudes of psychiatrists in India to psychiatrists in Switzerland, the authors were able to ascertain one potential way to improve adoption of Palliative Psychiatry by taking a more inclusive stance on which treatments would fall under palliation. The authors in this section each build on the BPS approach as the rationale and scaffolding for development of healthcare interventions that live at the borders of traditional conceptualizations or accepted biomedical treatment.

## Teamwork and the BPS approach

The evolution of implementing a BPS approach led inevitably to teamwork. In practice, clinicians may become adept at assessing the biological, psychological and social components of health, yet patients with more complex BPS needs might benefit from collaboration of experts in the biological, psychological and social spheres of healthcare. Xiao et al. note that, despite wide-spread knowledge of the importance of the BPS approach, including in China, there is still little known about its practical implementation. Their mixed-methods study aimed to assess how the BPS approach is applied in a large tertiary hospital in China. They found that despite an interest in the BPS approach, few clinicians are incorporating the three essential components of the BPS approach in practice; they point to the lack of team collaboration or integration of biomedical and psychosocial expertise as being one likely barrier.

Several other papers in this Research Topic build on the science of teamwork (12, 13), and argue for using expertise from various disciplines to create a comprehensive plan for complex challenges. For example, a team might include an internal medicine physician bringing a biomedical perspective, a psychologist attending to the cognitive and/or emotional perspective, and a social worker addressing myriad social and logistic dimensions of the situation. Ideally each team member is grounded in a general biopsychosocial approach so they have this overall shared mental model as well as their own specific area of expertise. These papers raise issues about teamwork such as the importance of role differentiation and the need to develop a new “shared dedication” or “solidarity” across disciplines. Sunder et al. describe a team approach to providing BPS care while maintaining continuity of community mental health care in rural India during the pandemic lockdown. They note that the pivot to remote care led to increased reliance on technology and a subsequent shift in team dynamics and wellbeing. Noting the similar challenges faced by a US-based community mental health clinic during the pandemic, Lamberti wrote a commentary commending Sunder et al. on engaging new team members in the community “to build confidence and trust in healthcare professionals.” Poleshuck et al. also emphasize the advantages of engaging community members in the development of a novel collaborative team-based approach to intimate partner violence. They discuss the advantage of convening bi-weekly multidisciplinary meetings and utilizing support from a community advisory board to ensure shared goals and purpose. Murphy et al. describe a BPS informed team approach to address cardiovascular risk among patients with severe mental illnesses. Using an illustrative case-example, they note the importance of regular communication and shared goal setting to ensuring accountability of various team-members.

## Building the foundation: Training innovations in the biopsychosocial approach

Vital to any approach to implementing the BPS approach is the way we prepare ourselves and others to do the work. Training, while historically focused deeply on discipline-specific content, skills, knowledge base, and intervention, increasingly represents an important milieu in which to plant seeds for the BPS approach, and for making room within those specific interventions, for example, for considering how these domains all influence patient and family outcomes. Rosenberg and Mullin (14) describe in depth some of the foundational skills in integration that can be incorporated into training across the professional lifespan, including in early phases of identity development and professionalization, and professional and Interprofessional continuing education throughout our careers. Funderburk et al. articulate this opportunity beautifully in their paper describing the potency of shared medical visits, conjoint appointments, and pre-session huddles in integrated primary care settings.

Training ideally focuses on core competencies of integration. These may start as additive to the discipline-specific content, but hopefully become foundational over time. Beginning with a solid curriculum rooted in the biopsychosocial approach is vital, and from that foundation, the BPS approach can propel learning around complex social systems like institutionalized racism and health disparities. Sanders and Fiscella. offer a particularly sobering and instructive application of the BPS approach to teaching through an antiracist lens for clinicians serving already marginalized populations. Their work challenges us to consider the urgent need for all of us to incorporate health equity principles into our teaching and practice.

As clearly evidenced by the last several years of the pandemic, novel approaches to delivery of this training must be considered, including ones that seize opportunities to train health professionals across discipline boundaries and in venues that allow for easier access. Gils et al. describe their innovative approach to building skills to treat and support patients with persistent somatic symptoms. Their online “e-learning” modules demonstrate the feasibility of providing highly satisfying training in groups for participants across health disciplines. The potential impact of such training platforms in this endemic phase of COVID is quite clear, including ones that leverage teams and learning across professional/role-related boundaries.

The stark rise in opioid-related deaths in the US since the start of the pandemic has been a clear example of the need for more biopsychosocially-attuned clinicians and staff across the spectrum of healthcare settings, not just in substance use care facilities. Russell et al. share an innovative approach to expanding access through integrating BPS training into buprenorphine training, an intervention that not only opens more doors to care, but also mitigates clinicians' likelihood to apply a moral lens to patient care and to hold hope that those suffering with addiction can be effectively treated.

The COVID pandemic has only served to highlight the deleterious effects of isolation and insufficient social support for those navigating the health system and dealing with disease. The crisis brought about by the inability of our existing systems of care to meet the exponentially rising demand and need have served as an urgent reminder that health is a product of the social and contextual circumstances in which humans live, in addition to the interpersonal and psychological responses to biological conditions. This Research Topic reveals both the need and the creativity of a variety of biopsychosocial approaches to these and the many other complex health challenges experienced around the world today.

## Author contributions

All authors listed have made a substantial, direct, and intellectual contribution to the work and approved it for publication.

## Conflict of interest

The authors declare that the research was conducted in the absence of any commercial or financial relationships that could be construed as a potential conflict of interest.

## Publisher's note

All claims expressed in this article are solely those of the authors and do not necessarily represent those of their affiliated organizations, or those of the publisher, the editors and the reviewers. Any product that may be evaluated in this article, or claim that may be made by its manufacturer, is not guaranteed or endorsed by the publisher.
